# A Manual of Dermatology

**Published:** 1885-05

**Authors:** 


**A Manual of Dermatology.** By A. R. ROBINSON, M. B., L. R. C. P., Professor of Dermatology New York Polyclinic, etc. New York: Bermingham & Co., 28 Union Square.

Of the making of books upon diseases of the skin, there would appear to be no end. Their number, however, is an index of the especial study which the cutaneous diseases have, of late years, received. The present book is by a distinguished author, but is intended to be the basis of a larger and more original work. It is a concise and fairly complete description of the different diseases, comprised in a handsome volume of nearly 700 pages. The publishers have issued it in handsome form.

